# Estrogen Receptor-Related DNA and Histone Methylation May Be Involved in the Transgenerational Disruption in Spermatogenesis by Selective Toxic Chemicals

**DOI:** 10.3389/fphar.2019.01012

**Published:** 2019-09-11

**Authors:** Xiao Han, Pengfei Zhang, Wei Shen, Yong Zhao, Hongfu Zhang

**Affiliations:** ^1^College of Animal Science and Technology, Qingdao Agricultural University, Qingdao, China; ^2^College of Life Sciences, Qingdao Agricultural University, Qingdao, China; ^3^State Key Laboratory of Animal Nutrition, Institute of Animal Sciences, Chinese Academy of Agricultural Sciences, Beijing, China

**Keywords:** H_2_S, NH_3_, spermatogenesis, transgenerational, DNA methylation, histone methylation, estrogen signaling

## Abstract

Air pollution is a global threat to human health especially spermatogenesis. Animal and epidemiological studies suggest that epigenetic factors can transmit the pathologies transgenerationally. Paternal epigenetic effects can greatly impact offspring health. In this study and together with our previous report, we found that H_2_S donor Na_2_S and/or NH_3_ donor NH_4_Cl diminished mouse fertility, decreased spermatozoa concentration and motility, and impaired spermatogenesis in three consequent generations (F0, F1, and F2). In the current study, we found that DNA methylation, histone methylation, and estrogen receptor alpha (ERα) were impaired by NH_4_Cl and/or Na_2_S in F0, F1, and F2 mouse testes. Moreover, NH_4_Cl and/or Na_2_S might act as environmental endocrine-disrupting chemicals to decrease estrogen and testosterone in mouse blood. It has been reported that ERα signaling is intertwined together with DNA methylation and histone methylation, which plays very important roles in spermatogenesis. These data together indicate that the transgenerational disruption in spermatogenesis by NH_4_Cl and/or Na_2_S may be through ERα-related DNA methylation and histone methylation pathways. Therefore, we strongly recommend that greater attention should be paid to NH_3_ and/or H_2_S contamination to minimize their impact on human health especially spermatogenesis.

## Introduction

Air pollution is a global threat to human health (Shukla et al., 2019). The infertility rate has been significantly elevated from 7% to 8% in 1960 to a current level of 20%–35% due to the decrease in the sperm concentration and motility (Frutos et al., 2015; Wijesekara et al., 2015; Checa Vizcaíno et al., 2016; Levine et al., 2017). Many investigations found that air pollution has played a very vital role in the decrease in sperm quality and fertility (Frutos et al., 2015; Wijesekara et al., 2015; Checa Vizcaíno et al., 2016). Air pollution includes gaseous and particulate matter (PM) in a variety of formats (Samet et al., 2004), and it comes from the increasing emissions from vehicles, industry, power stations, and other sources (Samet et al., 2004; Frutos et al., 2015; Wijesekara et al., 2015; Checa Vizcaíno et al., 2016). Motor vehicle exhaust, including a variety of toxic components (carbon monoxide, nitrogen oxides, volatile organic compounds, ozone, PM, and polycyclic aromatic hydrocarbons), has been reported to pose great adverse impacts on spermatogenesis and testosterone synthesis (Rengaraj et al., 2015). There are many kinds of volatile organic compounds in air pollution such as ozone, carbon monoxide (CO), sulfur dioxide (SO_2_), nitrogen oxides (NO_x_), ammonia (NH_3_), hydrogen sulfide (H_2_S), and others that are free or bound to air PM. Moreover, there are lots of sulfates (SO_4_^2−^), nitrates (NO_3_^−^), and ammonium (NH_4_^+^) in PM2.5 (PM <2.5 mm in diameter) (12). At the same time, PM can carry a large amount of NH_3 _and H_2_S (up to 7 μg NH_3_ per mg of respirable PM) (Samet et al., 2004; Cambra-López et al., 2010).

Epigenetic factors have been demonstrated to be disrupted by environmental contaminations. These altered epigenetic marks are embodied in the developing male germ cells and passed on to offspring to influence the health and development of offspring (Wu et al., 2015). DNA methylation, histone modifications, noncoding RNAs, and protamine code are the main epigenetic factors (Carrell 2012; Gannon et al., 2014). Epigenetic modification has been reported to play vital roles in spermatogenesis (Ge et al., 2017). The abnormal epigenetic alterations in spermatogenesis can decrease sperm quality and consequently result in male infertility, and many diseases in offspring (Aston et al., 2012).

During the past decade, the interest has been increased in the field of sperm epigenetics and there has been progress in this research field. Many investigations have reported that DNA methylation, histone posttranslational modifications, and non-coding RNAs play vital roles in sperm epigenetic states. Moreover, spermatozoon has been revealed to have a unique epigenome, and the chromatin regulation during male germline development is very complex (Champroux et al., 2018; Illum et al., 2018). Environmental exposures and paternal lifestyle have been found to modify the sperm epigenome, which in turn disrupts the health of future generations (Champroux et al., 2018). Germline epigenetic alterations induced by environmental contaminations may cause the aberrant gene expression and disease generationally (Nilsson and Skinner, 2015). p,p’-DDE, has been reported to induce transgenerational impairment in spermatogenesis (Song and Yang, 2018), and air pollution (gases, or particle matters) caused transgenerational inheritance in many diseases (Shukla et al., 2019).

During the pubertal period, because of the spermatogonial proliferation, the expansion of meiotic and haploid germ cells, and the increase in the somatic cells (Sertoli cells and Leydig cells), the testes undergo a rapid growth, which makes this special period for the male reproductive system development (Koskenniemi et al., 2017; Zhang et al., 2019). In our previous study (Zhang et al., 2018), we have found that pubertal exposure to NH_4_Cl and/or Na_2_S decreased mouse sperm quality (reduction in sperm motility and concentration) and diminished male fertility, which can be passed into future generations (at least three generations). Moreover, the spermatogenesis was disrupted in F0 and F1 generations of mice with impairment in many signaling pathways in spermatogenesis (Zhang et al., 2018). However, it is unknown how the disruption in spermatogenesis by NH_4_Cl and/or Na_2_S is transgenerational. Early this year, Shukla et al. reported that air pollution (gases or particle matters) can cause transgenerational inheritance in many diseases by the transmission of epimutations from gametes to zygotes (Shukla et al., 2019). The objectives of this investigation were designed to explore the underlying epigenetic mechanisms of the transgenerational effects of H_2_S and/or NH_3_ on spermatogenesis.

## Materials and Methods (Detailed Materials and Methods in Supplementary Information)

**Study design**. This investigation was carried out in strict accordance with the recommendations in the Guide for the Care and Use of Laboratory Animals of the National Institutes of Health. *The protocol in this investigation has been approved by the Committee on the Ethics of Animal Experiments of* Qingdao Agricultural University IACUC (Institutional Animal Care and Use Committee) (Zhang et al., 2018). Mice were raised under the following conditions: a 12-h light/12-h dark cycle, a temperature of 23°C, and a humidity of 50%–70%. Animals were handled humanely during the experiments. In order to minimize fighting, two animals were raised in each cage with a solid floor and woodchip bedding. Mice can access food (chow diet) and water constantly, and bedding was changed every other day (Zhang et al., 2018).

The main purpose of this investigation was to explore the epigenetic mechanisms of H_2_S and/or NH_3_ disruption on spermatogenesis and male fertility. Due to the gas phase of H_2_S and NH_3_, they can induce the irritant reactions or other side effects through pulmonary administration, and it is very hard to effectively control the doses. It has been reported that NH_4_Cl and Na_2_S can be used as the donors for NH_3_ and H_2_S in animal studies (Nowik et al., 2010; Hine et al., 2015). Therefore, Na_2_S and NH_4_Cl were used as the donor for H_2_S and NH_3_, respectively, in these investigations. ICR male mice (F0) were exposed to NH_4_Cl and/or Na_2_S *via* oral gavage. The NH_4_Cl and/or Na_2_S dosing solution was freshly prepared on a daily basis in phosphate-buffered saline (PBS) solution and administered as previously described (Hine et al., 2015; Zhang et al., 2018). The volume of gavage was 0.1 ml/mouse/day (Zhao et al., 2016; Zhang et al., 2018). The gavage took place every morning for 5 weeks starting at 25 days of age. Subsequently, 30 mice/treatment were humanely terminated for the analysis of spermatozoa quality and other parameters. A further 30 mice/treatment from each treatment were mated with normal (untreated) ICR female mice (male:female; 1:2). After the birth of the F1 litter, the number of live pups/litter was counted and all mice were raised similarly without further treatment (normal condition). At the age of 8 weeks (F1), 30 male mice/treatment were humanely terminated for analysis of spermatozoa quality and other parameters. A further 30 male mice/treatment were mated with normal ICR female mice (male/female, 1:2) and subsequently underwent a similar procedure. After the birth of the F2 litter, the number of live pups/litter was counted and all mice were raised in a similar manner without further treatment (study scheme in **Figure S1**). At the age of 8 weeks (F2), 30 male mice/treatment were humanely terminated for analysis of spermatozoa quality and other parameters.

### Evaluation of Spermatozoa Motility Using a Computer-Assisted Sperm Analysis System

Spermatozoa motility was assessed by a computer-assisted sperm assay (CASA) method according to World Health Organization guidelines (WHO, 2010; Zhang et al., 2018).

### Morphological Observations of Spermatozoa

The extracted murine caudal epididymides were placed in RPMI, finely chopped, and subsequently Eosin Y (1%) was added for staining as described by Shin et al. (2009) and Zhang et al. (2018).

### Assessment of Acrosome Integrity

Acrosomal integrity was assayed by an intense staining on the anterior region of the sperm head under bright-field microscopy (AH3-RFCA, Olympus, Tokyo, Japan) and scored for intensity of acrosomal staining (Elangovan et al., 2006; Zhang et al., 2018).

### Detection of Protein Levels and Location in Testis by Immunofluorescent Staining

The methodology for immunofluorescent staining of testicular samples was reported in our recent publication (Wang et al., 2016; Zhang et al., 2018) (**Table S1** for primary antibody information). A minimum of 1000 cells were counted for each sample of each experiment. Then, the data were normalized to control.

### Statistical Analysis

Quantitative data were presented as the mean ± SEM. The data were statistically analyzed by SPSS statistical software (IBM Co., NY). Statistical analysis of data was carried out by one-way analysis of variance (ANOVA), followed by LSD multiple comparison test. All treatment groups were compared with each other for every parameter. Differences were considered statistically significant if the *p* value was less than 0.05.

## Results

### Na_2_S and/or NH_4_Cl Reduced Mouse Sperm Quality

**Figure 1:**
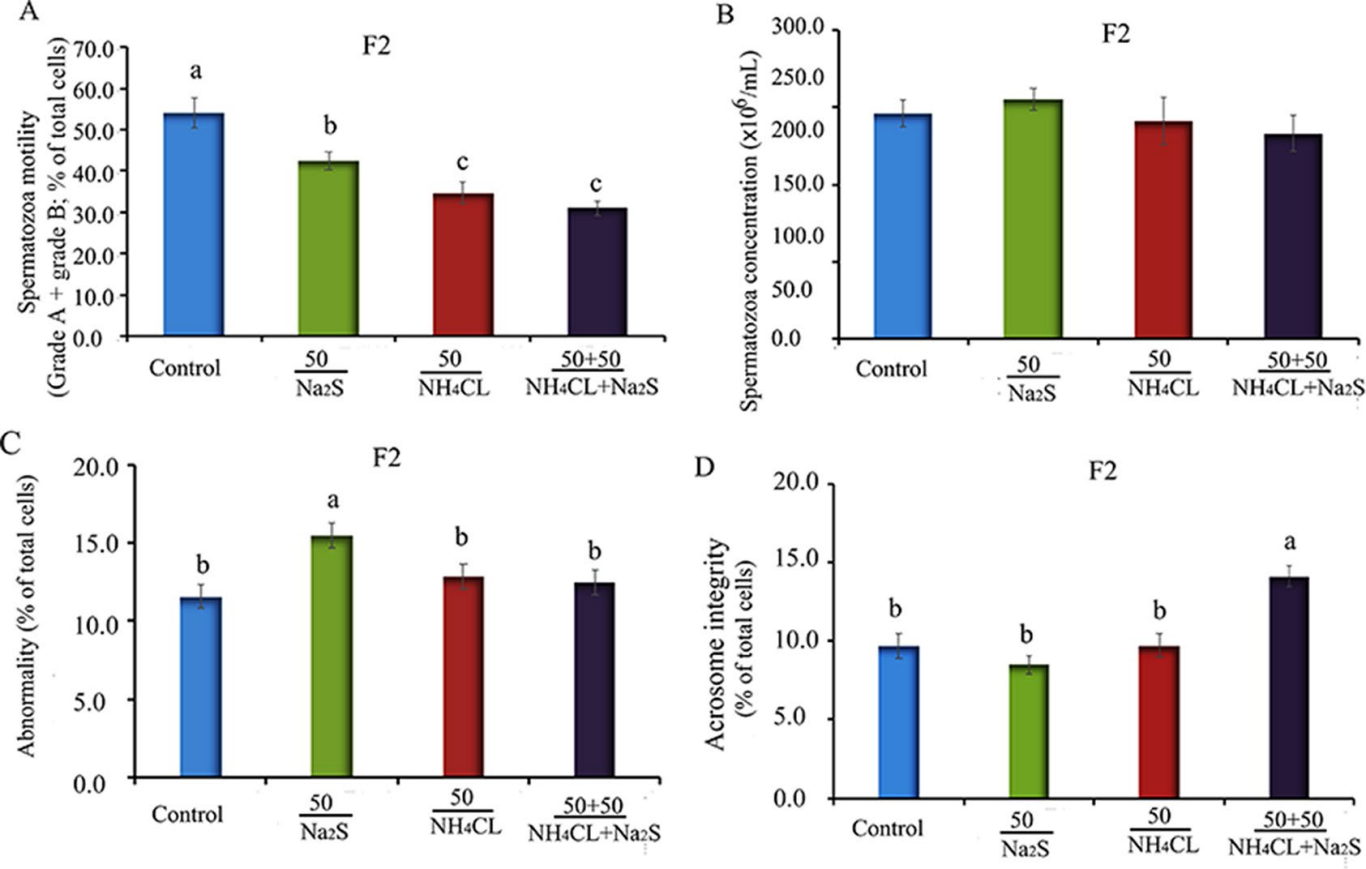
**(A)** F2 mice sperm motility. Y-axis = percentage of total cells, X-axis = the treatment concentration (mg/kg body weight). **(B)** F2 mouse sperm concentration. Y-axis = number of cells, X-axis = the treatment concentration (mg/kg body weight). **(C)** The abnormality of F2 mouse spermatozoa detected by eosin Y staining. **(D)** The abnormal acrosome integrity of F2 mouse spermatozoa detected by commas blue staining. N > 6. a, b, c indicate a significant difference among different treatments (p < 0.05).

In our previous study (Zhang et al., 2018), it has been reported that mouse (F0) sperm motility and concentration were decreased by Na_2_S and/or NH_4_Cl (**Figure S2**) after a 5-week exposure. It was found that sperm motility and concentration were decreased by Na_2_S and/or NH_4_Cl exposure in F1 mice (**Figure S3**). Moreover, in the current study, it was found that sperm motility was decreased by Na_2_S-50 mg/kg, NH_4_Cl-50 mg/kg, and NH_4_Cl-­50 mg/kg + Na_2_S-50 mg/kg exposure (**Figure 1A**); however, sperm concentration was not altered (**Figure 1B**) in F2 mice. Sperm abnormality and sperm acrosome integrity were changed a little by Na_2_S-50 mg/kg or NH_4_Cl-50 mg/kg + Na_2_S-50 mg/kg exposure in F2 mice (**Figures 1C, D**). Mouse body weight and organ indexes were not changed in all treatments (**Table 1** for F2 mice; **Table S2** for F0 and F1 mice).

**Table 1:** Body parameters of F2 mice. Data are present as average ± SEM.

		Na_2_S	NH_4_Cl	NH_4_Cl+Na_2_S
	Control	50	50	50 + 50
F2				
Body weight (g)	32.4 ± 2.4	32.6 ± 2.1	32.5 ± 2.0	30.1 ± 2.8
Liver organ index (% of body weight)	5.3 ± 0.3	6.4 ± 0.7	6.0 ± 0.7	4.9 ± 0.7
Spleen organ index (% of body weight)	0.8 ± 0.1	0.6 ± 0.0	0.9 ± 0.0	0.5 ± 0.0
Kidney organ index (% of body weight)	1.6 ± 0.1	1.7 ± 0.1	1.7 ± 0.1	1.5 ± 0.1
Testis organ index (% of body weight)	0.8 ± 0.1	0.7 ± 0.1	0.8 ± 0.0	0.8 ± 0.1

### Na_2_S and/or NH_4_Cl Impaired DNA Methylation Status in Murine (F0, F1, and F2) Testes

**Figure 2:**
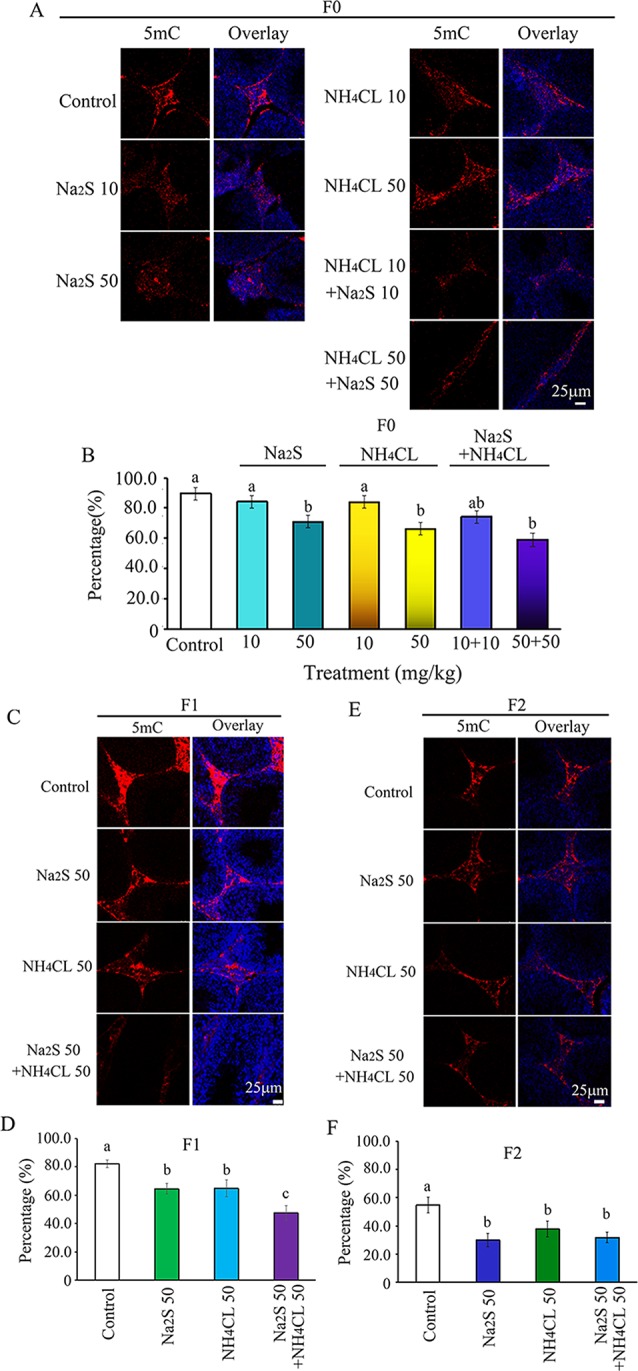
Protein 5mC in mouse testicular tissue as detected by immunofluorescence staining. **(A)** 5mC staining in F0 mouse testis. **(B)** Quantitative data for 5mC in F0 mouse testis. **(C)** 5mC staining in F1 mouse testis. **(D)** Quantitative data for 5mC in F1 mouse testis. **(E)** 5mC staining in F2 mouse testis. **(F)** Quantitative data for 5mC in F2 mouse testis. N > 6. a, b, c indicate a significant difference among different treatments (p < 0.05).

It has been reported that DNA methylation markers 5mC and 5hmC are very important factors in regulation of spermatogenesis (Gan et al., 2013; Hackett et al., 2013). DNA methylation markers 5mC and 5hmC were determined in the mouse testes in this study. It was found that 5mC was mainly expressed in leydig cells (Zhang et al., 2019). The number of 5mC positive leydig cells in F0 mouse testes was significantly decreased by Na_2_S-50 mg/kg, NH_4_Cl-50 mg/kg, and NH_4_Cl-50 mg/kg + Na_2_S-50 mg/kg exposure (**Figures 2A, B**). Interestingly, 5mC positive leydig cells in F1 and F2 generation mouse testes were also significantly decreased by Na_2_S-50 mg/kg, NH_4_Cl-50 mg/kg, and NH_4_Cl-50 mg/kg + Na_2_S-50 mg/kg exposure (**Figures 2C–F**).

**Figure 3:**
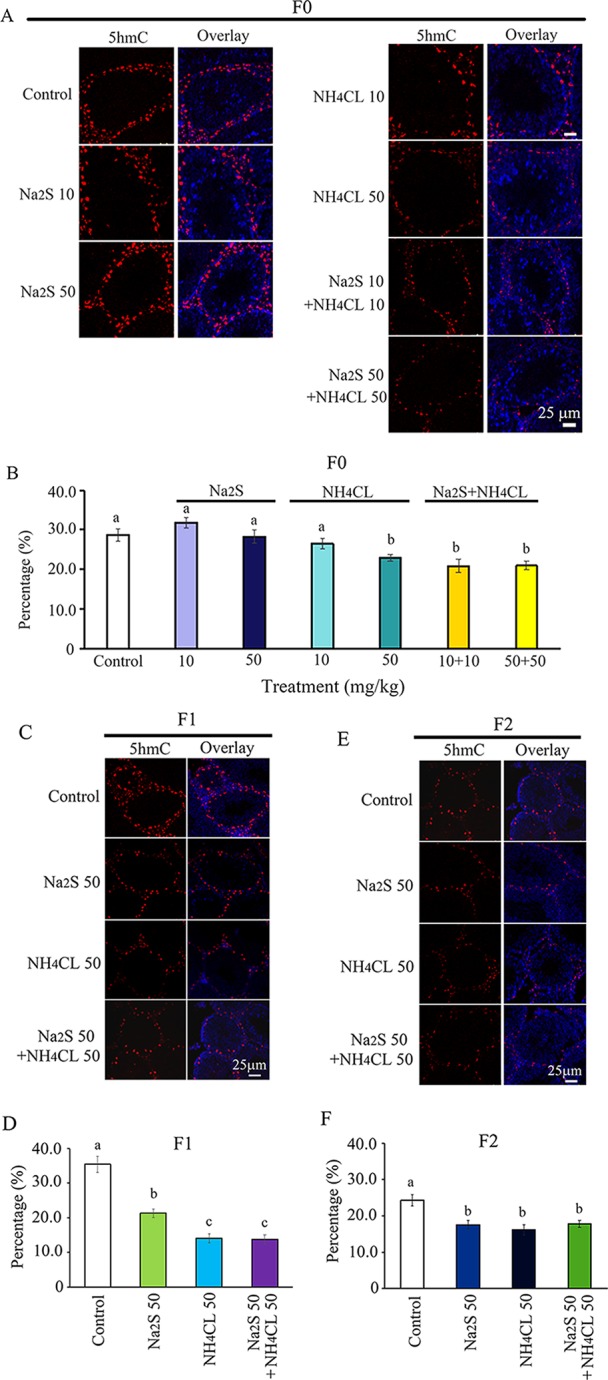
Protein 5hmC in mouse testicular tissue as detected by immunofluorescence staining. **(A)** 5hmC staining in F0 mouse testis. **(B)** Quantitative data for 5hmC in F0 mouse testis. **(C)** 5hmC staining in F1 mouse testis. **(D)** Quantitative data for 5hmC in F1 mouse testis. **(E)** 5hmC staining in F2 mouse testis. **(F)** Quantitative data for 5hmC in F2 mouse testis. N > 6. a, b, c indicate a significant difference among different treatments (p < 0.05).

Moreover, we found that 5hmC was mainly expressed in spermatogonia stem cells (SSCs; Men et al., 2019). The number of 5hmC-positive SSCs in F0 mouse testes was significantly diminished by NH_4_Cl-50mg/kg, NH_4_Cl-10mg/kg + Na_2_S-10mg/kg, and NH_4_Cl-50 mg/kg + Na_2_S-50 mg/kg exposure (**Figures 3A, B**). 5hmC positive SSCs in F1 and F2 generation mouse testes were significantly reduced by Na_2_S-50 mg/kg, NH_4_Cl-50 mg/kg, and NH_4_CL-50 mg/kg + Na_2_S-50 mg/kg exposure (**Figures 3C–F**). The data suggest that DNA methylation status in mouse testes is disrupted by NH_4_Cl and/or Na_2_S exposure, which can be passed on to future generations (transgenerational).

### Na_2_S and/or NH_4_Cl Disrupted Histone Methylation in Murine (F0, F1, and F2) Testes

**Figure 4:**
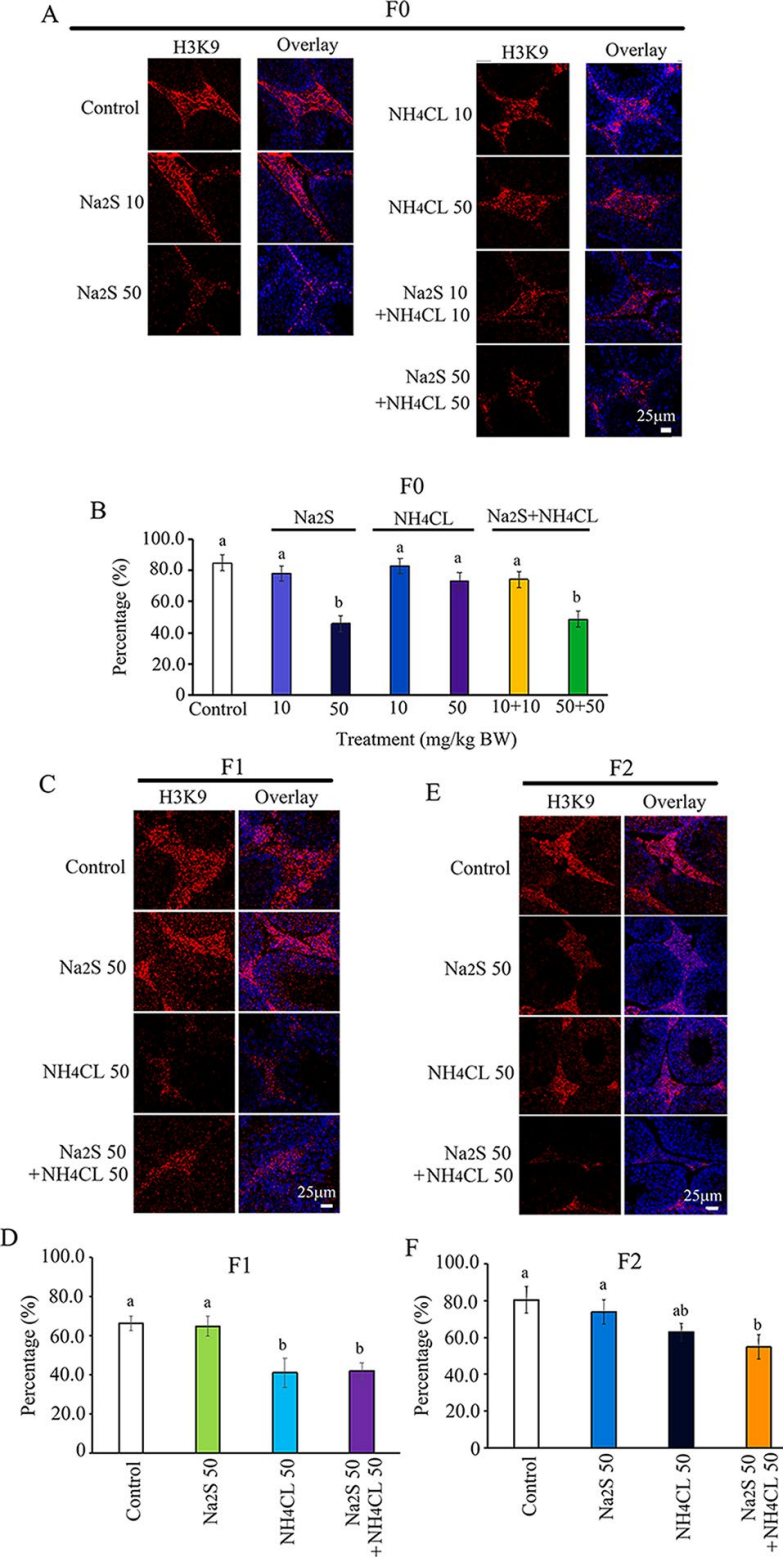
Protein H3K9 in mouse testicular tissue as detected by immunofluorescence staining. **(A)** H3K9 staining in F0 mouse testis. **(B)** Quantitative data for H3K9 in F0 mouse testis. **(C)** H3K9 staining in F1 mouse testis. **(D)** Quantitative data for H3K9 in F1 mouse testis. **(E)** H3K9 staining in F2 mouse testis. **(F)** Quantitative data for H3K9 in F2 mouse testis. N > 6. a, b indicate a significant difference among different treatments (p < 0.05).

Histone methylation markers H3K9 and H3K27 have been reported to play important roles in spermatogenesis (Ebert et al., 2006; Choi et al., 2013; Ge et al., 2017). Histone methylation markers H3K9 and H3K27 were analyzed in the mouse testes in this study. H3K9 was mainly expressed in leydig cells (Choi et al., 2013; Alamdar et al., 2017). The number of H3K9-positive leydig cells was significantly decreased by Na_2_S-50 mg/kg and NH_4_Cl-50 mg/kg + Na_2_S-50 mg/kg exposure in F0 mouse testes (**Figures 4A, B**). H3K9-positive leydig cells in F1 and F2 mouse testes were significantly reduced by NH_4_Cl-50 mg/kg + Na_2_S-50 mg/kg exposure (**Figures 4C–F**).

**Figure 5:**
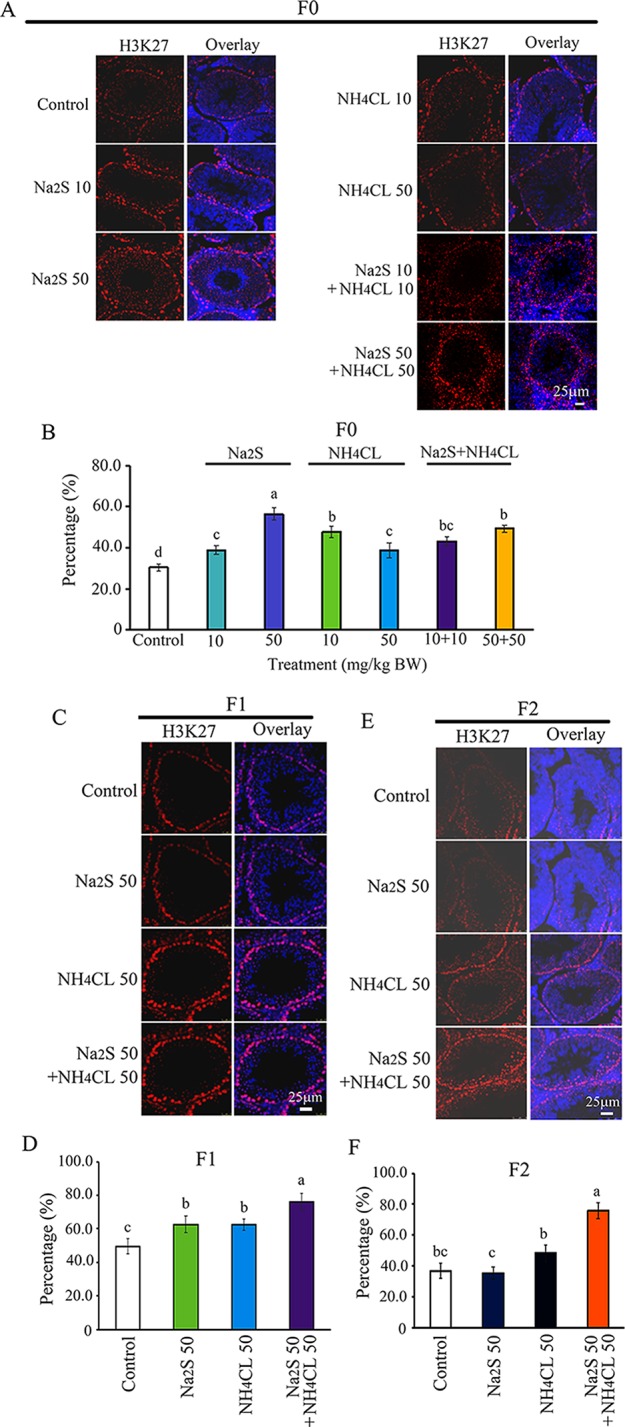
Protein H3K27 in mouse testicular tissue as detected by immunofluorescence staining. **(A)** H3K27 staining in F0 mouse testis. **(B)** Quantitative data for H3K27 in F0 mouse testis. **(C)** H3K27 staining in F1 mouse testis. **(D)** Quantitative data for H3K27 in F1 mouse testis. **(E)** H3K27 staining in F2 mouse testis. **(F)** Quantitative data for H3K27 in F2 mouse testis. N > 6. a, b, c indicate a significant difference among different treatments (p < 0.05).

Furthermore, H3K27 was mainly expressed in SSCs (Zhang et al., 2019). The number of H3K27-positive SSCs in F0 mouse testes was significantly increased by Na_2_S-10mg/kg, Na_2_S-50mg/kg, NH_4_Cl-10mg/kg, NH_4_Cl-50mg/kg, NH_4_CL-10mg/kg + NH_4_Cl-10mg/kg, and NH_4_CL-50 mg/kg + Na_2_S-50 mg/kg exposure (**Figures 5A, B**). H3K27-positive SSCs in F1 and F2 mouse testes were significantly elevated by NH_4_Cl-50 mg/kg + Na_2_S-50 mg/kg exposure (**Figures 5C–F**). The data suggest that histone methylation status in mouse testes is impaired by NH_3_ and/or H_2_S exposure, which can be inherited (transgenerational).

### Na_2_S and/or NH_4_Cl Damaged Estrogen Receptor Alpha (ER**α**) in Murine (F0, F1, and F2) Testes

**Figure 6:**
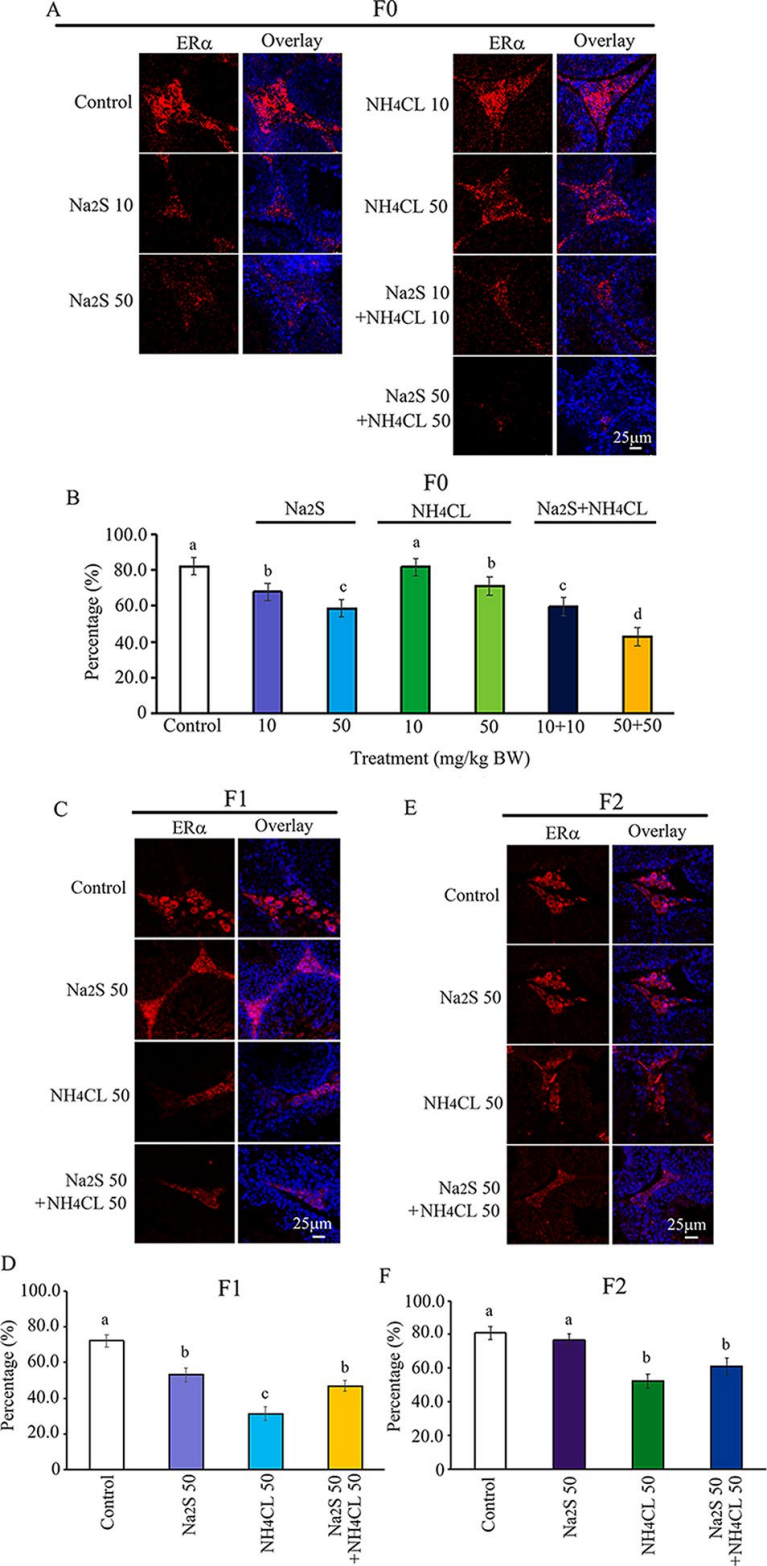
Protein ERα in mouse testicular tissue as detected by immunofluorescence staining. **(A)** ERα staining in F0 mouse testis. **(B)** Quantitative data for ERα in F0 mouse testis. **(C)** ERα staining in F1 mouse testis. **(D)** Quantitative data for ERα in F1 mouse testis. **(E)** ERα staining in F2 mouse testis. **(F)** Quantitative data for ERα in F2 mouse testis. N > 6. a, b, c indicate a significant difference among different treatments (p < 0.05).

The ERα pathway has been found to play vital roles in spermatogenesis (Carreau et al., 2011; Cooke et al., 2017). The interaction of ERα with DNA methylation or histone methylation has also been reported to be very important in spermatogenesis (Marques et al., 2013; Dumasia et al., 2017). In the current study, the expression of ERα was determined in F0, F1, and F2 mouse testes. It was found that ERα was mainly expressed in leydig cells. The number of ERα-positive leydig cells was significantly diminished by Na_2_S-10 mg/kg, Na_2_S-50 mg/kg, NH_4_Cl-50 mg/kg, NH_4_Cl-10 mg/kg + Na_2_S-10 mg/kg, and NH_4_Cl-50 mg/kg + Na_2_S-50 mg/kg exposure in F0 mouse testes dose dependently (**Figures 6A, B**). ERα-positive leydig cells in F1 and F2 mouse testes were significantly reduced by NH_4_Cl-50 mg/kg, Na_2_S-50 mg/kg, and NH_4_Cl-50 mg/kg + Na_2_S-50 mg/kg exposure (**Figures 6C–F**). The result here indicates that the ERα pathway in mouse testes is disrupted by NH_3_ and/or H_2_S exposure, which can be passed on to future generations as well (transgenerational).

## Discussion

In this study and together with our previous reports (Zhao et al., 2016; Zhang et al., 2018), we found that H_2_S donor Na_2_S and/or NH_3_ donor NH_4_Cl diminished mouse fertility, decreased spermatozoa concentration and motility, and impaired spermatogenesis in three consequent generations (F0, F1, and F2 mice). Spermatogenesis has been found to be very sensitive to environment contamination such as pesticides, waste, air PM, and reactive gas NH_3_, H_2_S, and others. Moreover, environmental contamination has been reported to disrupt the epigenetic factors. These disrupted epigenetic factors can act as epigenetic markers embodied within the developing male germ cells, and can be passed on to offspring to disrupt offspring health and development (Wu et al., 2015).

In human civilization, environment has been damaged incontrovertibly, and air pollution has become a global threat for human health and especially reproductive health (Shukla et al., 2019). It was reported by World Health Organization (WHO) that 95% of human beings are living with an unhealthy lifestyle (WHO, 2016; Landrigan et al., 2018). It has been reported by epidemiological and clinical studies that air pollution is connected with various diseases including cardiovascular diseases, respiratory diseases, cancer, and reproductive diseases (Yang et al., 2017; Huang et al., 2018). It has been found that motor vehicle exhaust disrupted male reproductive functions through altering organ weight, decreasing the sperm quality, and promoting oxidative stress (Rengaraj et al., 2015). Air pollutant has adverse impacts on sperm motility and male fertility (Centola et al., 2016; Deng et al., 2016). Airborne PMs have been found to be very dangerous to human health (Samet et al., 2004; Somers et al., 2004). NH_3_ and H_2_S are present as gaseous components of air pollution where they are abundant and reactive because NH_3_ is the main component of total reactive nitrogen and H_2_S is the predominant sulfur contaminant of natural gas (Reiffenstein et al., 1992; Behera et al., 2013; Braissant et al., 2013; di Masi and Ascenzi, 2013). Moreover, NH_3_ and H_2_S can be bound to PMs or they can be free in the atmosphere.

Epigenetic modifications include DNA methylation, histone post-translational modification (PTM), and non-coding RNAs. DNA methylation is the most explored epigenetic modification compared to histone PTM or non-coding RNAs. DNA methylation plays vital roles in regulating gene expression globally (Champroux et al., 2018). Both 5-methylcytosine (5mC) and 5-hydroxymethylcytosine (5hmC) have been discovered to be most important DNA methylation markers (Gan et al., 2013; Hackett et al., 2013). Histone lysine (K) methylation is one of the most important histone PTMs because it is stable and inheritable. Histone methylation acts as transcriptional activation or repression (Ebert et al., 2006; Li et al., 2007; Brykczynska et al., 2010). It has been reported by animal studies and epidemiological reports that epigenetic factors can transmit the pathologies across generations, which is called epigenetic inheritance or transgenerational epigenetic inheritance. Recently, it was found that paternal epigenetics can greatly impact on the offspring health (Schaefer and Nadeau, 2015). In the current study, we found that DNA methylation and histone methylation were disrupted by NH_4_Cl and/or Na_2_S in F0 mouse testes. Moreover, the phenomena were found in F1 and F2 mouse testes, which indicated that NH_4_Cl and/or Na_2_S caused transgenerational epigenetic inheritance. It has been described by Martos et al. that impacts observed in the male germline during F2 generation can be transgenerational when it was induced during exposure to the adult male (F0) and his germline (F1) (Martos et al., 2015). In our studies, the F0 mice were exposed to NH_4_Cl and/or Na_2_S, then after exposure, the male mice were mated with normal female mice to generate F1 mice. F1 male mice were raised under normal conditions and mated with normal female mice to obtain F2 generation. Therefore, our findings of the disruption in spermatogenesis, DNA methylation, and histone methylation in F0, F1, and F2 mice are transgenerational.

Although NH_3_ and H_2_S have not been considered as environmental endocrine-disrupting chemicals (EDCs), we have found that NH_4_Cl and/or Na_2_S exposure decreased testosterone and estrogen in mouse plasma after a 5-week treatment (Zhang et al., 2018). The estrogen receptor signaling pathway plays vital roles in male reproductive development especially spermatogenesis (Couse et al., 1997; Hess et al., 1997; Carreau et al., 2011; Cooke et al., 2017). In the current investigation, we found that NH_4_Cl and/or Na_2_S exposure decreased the expression of estrogen receptor alpha (ERα) in F0 mouse testes; interestingly, the disruption in ERα was also found in F1 and F2 mouse testes. Our data indicate that NH_4_Cl and/or Na_2_S may act as EDCs to disrupt spermatogenesis, which can be transgenerational.

It has been reported that the ERα signaling pathway can regulate histone methylation during spermatogenesis (Dumasia et al., 2017). During mouse spermatogenesis, ERα agonist (4,4′,4″-(4-Propyl-[1H] pyrazole-1,3,5-triyl; PPT) increased H3K27me3 expression (Dumasia et al., 2017). Furthermore, estrogen signaling and DNA methylation are intertwined together tightly, which plays a very important role in spermatogenesis (Marques et al., 2013; Vrtačnik et al., 2014). In our current study, it was found that spermatogenesis was disrupted by NH_4_Cl and/or Na_2_S exposure; moreover, DNA methylation, histone methylation, and ERα were impaired by NH_4_Cl and/or Na_2_S in F0, F1, and F2 mice testes.

## Conclusion

In summary, the aims of our current study were to explore the epigenetic mechanisms of NH_4_Cl and/or Na_2_S, disrupting the spermatogenesis transgenerationally. It was demonstrated that DNA methylation, histone methylation, and ERα were impaired by NH_4_Cl and/or Na_2_S exposure in F0, F1, and F2 mouse testes. It has been reported that ERα pathway interacts with DNA methylation and histone methylation in spermatogenesis. These data together indicate that the transgenerational disruption in spermatogenesis by NH_4_Cl and/or Na_2_S might be through ERα-regulated DNA methylation and histone methylation. Therefore, we strongly recommend that greater attention should be paid to NH_4_Cl and/or Na_2_S contamination to minimize its impact on human health especially spermatogenesis.

## Data Availability

All datasets generated for this study are included in the manuscript/**Supplementary Files**.

## Ethics Statement

The animal study was reviewed and approved by Committee on the Ethics of Animal Experiments of Qingdao Agricultural University IACUC (Institutional Animal Care and Use Committee).

## Author Contributions

HZ, WS, and YZ provided key intellectual input in the conception and design of these studies and YZ wrote the manuscript. XH and PZ performed animal experiments and IHF experiments. HZ and WS contributed to the writing of the manuscript. All authors reviewed the manuscript.

## Funding

This study was supported by the National Natural Science Foundation of China (31772408) and the National Key Research and Development Program of China (2016YFD0500500).

## Conflict of Interest Statement

The authors declare that the research was conducted in the absence of any commercial or financial relationships that could be construed as a potential conflict of interest.
