# Supplementary figures and images for: Estrogen Receptor-Related DNA and Histone Methylation May Be Involved in the Transgenerational Disruption in Spermatogenesis by Selective Toxic Chemicals
Source: Front Pharmacol. 2019 Sep 11;10:1012. doi: 10.3389/fphar.2019.01012 (PMC6749155; doi:10.3389/fphar.2019.01012)

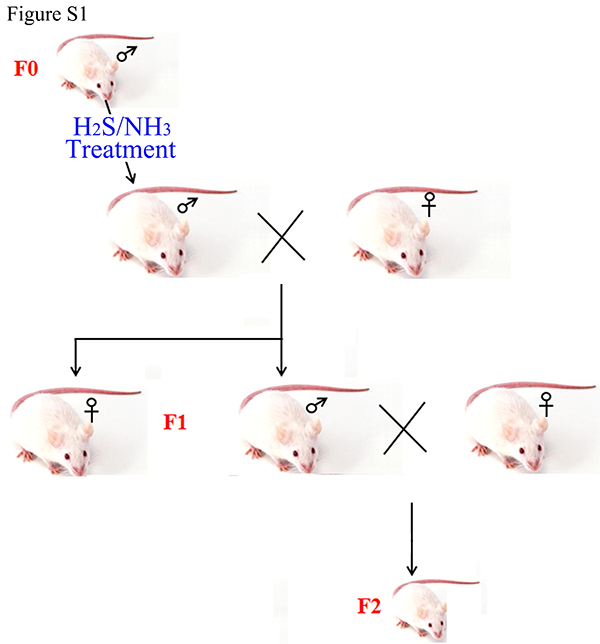

Supplement: Supplementary file 1 [file Image_1.tif]

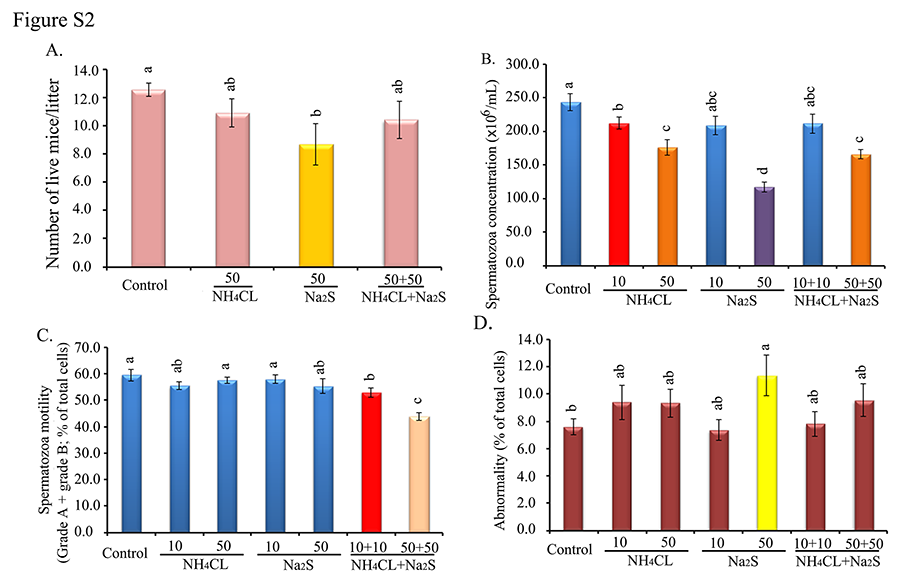

Supplement: Supplementary file 2 [file Image_2.tif]

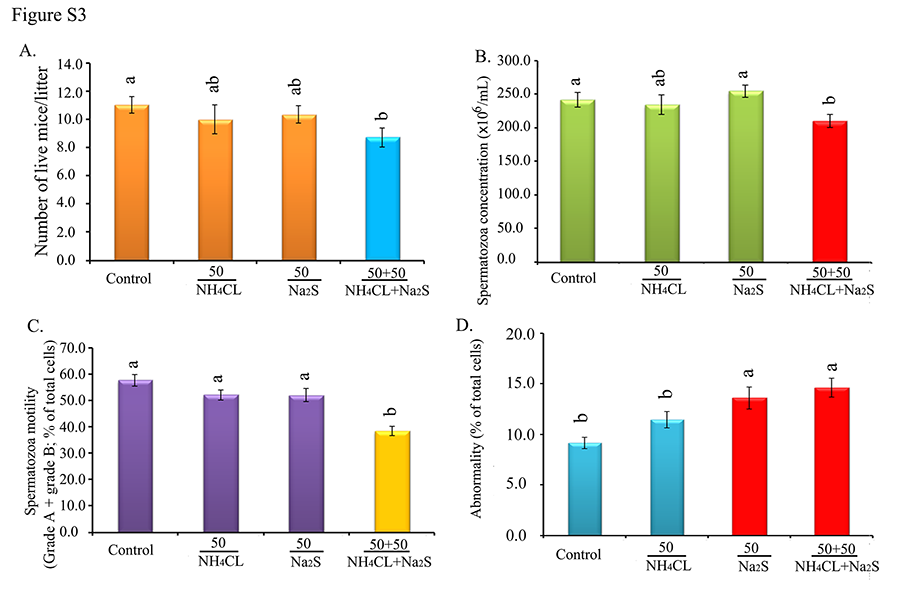

Supplement: Supplementary file 3 [file Image_3.tif]
