# Supplementary material for: Estrogen Receptor-Related DNA and Histone Methylation May Be Involved in the Transgenerational Disruption in Spermatogenesis by Selective Toxic Chemicals
Source: Front Pharmacol. 2019 Sep 11;10:1012. doi: 10.3389/fphar.2019.01012 (PMC6749155; doi:10.3389/fphar.2019.01012)
Supplement: Supplementary file 4 [file Table_1.doc]

**Table S1.** Primary antibody information

| **Gene symbol** | **Name** | **Cat. #** | **Predicted size** | **Source (Animal)** | **Company** |
| --- | --- | --- | --- | --- | --- |
| ERα | Estrogen and progesterone receptor | D8H8 | 66kDa | Rabbit (polyclonal) | Cell Signaling Technology CO. |
| H3K27me3 | Trimethyl histone H3 (Lys27) | ABE44 | 17kDa | Rabbit (polyclonal) | Merck Millipore Biotechnology CO. |
| H3K9me2 | Dimethyl histone H3 (Lys9) | 05-1249 | 17kDa | Mouse(monoclonal) | Merck Millipore Biotechnology CO. |
| 5mC | 5-methylcytosine (5-mC) | Ab10805 |  | Mouse (monoclonal) | Abcam |
| 5hmC | 5-Hydroxymethylcytosine (5-hmC) | 39791 |  | Rabbit | Activemotif Biotechnology CO. |
