# Supplementary material for: Estrogen Receptor-Related DNA and Histone Methylation May Be Involved in the Transgenerational Disruption in Spermatogenesis by Selective Toxic Chemicals
Source: Front Pharmacol. 2019 Sep 11;10:1012. doi: 10.3389/fphar.2019.01012 (PMC6749155; doi:10.3389/fphar.2019.01012)
Supplement: Supplementary file 5 [file Table_2.docx]

Table S2. Body and plasma parameters of F0 and F1 mice. Data present as Average ± SEM. a, b, c indicate a significant difference among different treatments (n>6; p < 0.05).

|  |  | **NH_4_CL** | | **Na_2_S** | | **NH_4_CL+Na_2_S** | |
| --- | --- | --- | --- | --- | --- | --- | --- |
|  | **Control** | **10** | **50** | **10** | **50** | **10+10** | **50+50** |
| **F0** |  |  |  |  |  |  |  |
| Body weight (g) | 34.6±1.0 | 35.1±1.0 | 34.1±0.7 | 34.3±0.9 | 34.1±1.1 | 33.3±1.2 | 32.2±0.8 |
| Liver organ index (% of body weight) | 5.3±0.2^a^ | 5.1±0.2^ab^ | 4.7±0.1^ab^ | 4.5±0.2^ab^ | 4. 5±0.1^b^ | 4.9±0.2^ab^ | 4.3±0.1^b^ |
| Spleen organ index (% of body weight) | 0.4±0.1 | 0.4±0.1 | 0.4±0.0 | 0.4±0.0 | 0.4±0.0 | 0.4±0.0 | 0.4±0.0 |
| Kidney organ index (% of body weight) | 1.5±0.1 | 1.6±0.0 | 1.6±0.1 | 1.5±0.1 | 1.5±0.1 | 1.5±0.1 | 1.5±0.1 |
| Testis organ index (% of body weight) | 0.8±0.1 | 0.8±0.0 | 0.8±0.0 | 0.8±0.0 | 0.8±0.0 | 0.8±0.0 | 0.8±0.0 |
|  |  |  |  |  |  |  |  |
| **F1** |  |  |  |  |  |  |  |
| Body weight (g) | 38.0±0.7 |  | 39.6±0.6 |  | 39.8±0.7 |  | 40.0±1.0 |
| Liver organ index (% of body weight) | 5.1±0.2^ab^ |  | 5.1±0.1^ab^ |  | 5.4±0.1^a^ |  | 5.0±0.1^b^ |
| Spleen organ index (% of body weight) | 0.4±0.0 |  | 0.4±0.0 |  | 0.4±0.0 |  | 0.3±0.0 |
| Kidney organ index (% of body weight) | 1.59±0.06^ab^ |  | 1.47±0.05^b^ |  | 1.64±0.04^a^ |  | 1.65±0.06^a^ |
| Testis organ index (% of body weight) | 0.8±0.0 |  | 0.8±0.0 |  | 0.8±0.0 |  | 0.8±0.0 |
|  |  |  |  |  |  |  |  |
