# Supplementary material for: Estrogen Receptor-Related DNA and Histone Methylation May Be Involved in the Transgenerational Disruption in Spermatogenesis by Selective Toxic Chemicals
Source: Front Pharmacol. 2019 Sep 11;10:1012. doi: 10.3389/fphar.2019.01012 (PMC6749155; doi:10.3389/fphar.2019.01012)
Supplement: Supplementary file 6 [file Table_3.doc]

**Detailed Materials and methods**

**Study design.** The protocol in this investigation has been approved by the Committee on the Ethics of Animal Experiments of Qingdao Agricultural University IACUC (Institutional Animal Care and Use Committee) (Zhang et al., 2018). Mice were raised in a condition with the light: dark cycle of 12:12 h and the temperature of 23 ^o^C and humidity of 50%–70%. Animals were handled humanely during the experiments. In order to minimize fighting, two animals were raised in each cage with a solid floor and woodchip bedding. Mice can access to food (chow diet) and water constantly, and bedding was changed every other day (Zhang et al., 2018).

The main purpose of this investigation was to explore the epigenetic mechanisms of H_2_S and/or NH_3_ disruption on spermatogenesis and male fertility. Due to gas phase of H_2_S and NH_3_, they can induce the irritant reactions or other side effects though pulmonary administration and it is very hard to effectively control the doses. It has been reported that NH_4_Cl and Na_2_S can be used as the donors for animal studies (Hine et al., 2015; Nowik et al., 2010). Therefore, Na_2_S and NH_4_Cl were used as the donor for H_2_S and NH_3_, respectively in these investigations. ICR male mice (F0) were exposed to NH_4_Cl and/or Na_2_S via oral gavage. The NH_4_Cl and/or Na_2_S dosing solution was freshly prepared on a daily basis in phosphate buffered saline (PBS) solution and administered as previously described (Zhang et al., 2018; Hine et al., 2015). There were 7 treatments (60 mice/treatment): (1) vehicle control (PBS); (2) NH_4_Cl-10 mg/kg BW; (3) NH_4_Cl-50 mg/kg BW; (4) Na_2_S-10 mg/kg BW; (5) Na_2_S-50 mg/kg BW; (6) NH_4_Cl-10 mg/kg + Na_2_S-10 mg/kg BW; (7) NH_4_Cl-50 mg/kg BW + Na_2_S-50 mg/kg BW. The volume of gavage was 0.1 ml/mouse/day. The gavage took place every morning for five weeks starting at 25 d of age. Subsequently, 30 mice/treatment were humanely terminated for the analysis of spermatozoa quality and other parameters. A further 30 mice/treatment from treatments (1) the vehicle control (PBS); (3) NH_4_Cl-50 mg/kg BW; (5) Na_2_S-50 mg/kg BW; and (7) NH_4_Cl-50 mg/kg BW + Na_2_S-50 mg/kg BW were mated with normal (untreated) ICR female mice (male: female; 1:2). After birth of the F1 litter, the number of live pups/litter was counted and all mice were raised similarly without further treatment (normal condition). At the age of 8 weeks (F1), 30 male mice/treatment were humanely terminated for analysis of spermatozoa quality and other parameters. A further 30 male mice/treatment were mated with normal ICR female mice (male: female; 1:2) and subsequently underwent a similar procedure. After birth of the F2 litter, the number of live pups/litter was counted and all mice were raised in a similar manner without further treatment (study scheme in Figure S1). At the age of 8 weeks (F2), 30 male mice/treatment were humanely terminated for analysis of spermatozoa quality and other parameters.

**Evaluation of spermatozoa motility using a computer-assisted sperm analysis system**

Spermatozoa motility was assessed by a computer-assisted sperm assay (CASA) method according to World Health Organization guidelines (Zhang et al., 2018; WHO 2010). After euthanasia, spermatozoa were collected from the cauda epididymis of mice and suspended in DMEM/F12 medium with 10% FBS and incubated at 37.5 ^o^C for 30 min; samples were then placed in a pre-warmed counting chamber (Zhang et al., 2018). The Micropic Sperm class analyzer (CASA system) was used in this investigation. It was equipped with a 20-fold objective, a camera adaptor (Eclipse E200, Nicon, Japan), and a camera (acA780-75gc, Basler, Germany), and it was operated by an SCA sperm class analyser (MICROPTIC S.L.). The classification of sperm motility was as follows: grade A linear velocity >22 μm s^-1^; grade B <22 μm s^-1^ and curvilinear velocity >5 μm s^-1^; grade C curvilinear velocity <5 μm s^-1^; and grade D = immotile spermatozoa (WHO 2010). The spermatozoa motility data represent the just grade A + grade B since the spermatozoa in these two grade are considered as the functional ones (WHO 2010).

**Morphological observations of spermatozoa**

The extracted murine caudal epididymides were placed in RPMI, finely chopped and subsequently Eosin Y (1%) was added for staining as described by Shin (Shin et al., 2009). Spermatozoa abnormalities were then viewed using an optical microscope and were classified into head or tail morphological abnormalities: two heads, two tails, blunt hooks, and short tails (Shin et al., 2009). The examinations were repeated three times, and 500 spermatozoa per animal were scored.

**Assessment of acrosome integrity**

After harvest, mouse spermatozoa were incubated at 37.5 ℃ for 30 min, then a drop of sperm suspension was uniformly smeared on a clean glass slide. Smeared slides were air dried and incubated in methanol for 2 min for fixation. After fixation, the slides were washed with PBS three times. Assessment of an intact acrosome was accomplished by staining the sperm with 0.025% Coomassie brilliant blue G-250 in 40% methanol for 20 min at room temperature. The slides were then washed three times with PBS and mounted with 50% glycerol in PBS. Acrosomal integrity was assayed by an intense staining on the anterior region of the sperm head under bright-field microscopy (AH3-RFCA, Olympus, Tokyo, Japan) and scored for intensity of acrosomal staining (Elangovan et al., 2006).

**Detection of protein levels and location in testis by immunofluorescent staining**

The methodology for immunofluorescent staining of testicular samples was reported in our recent publication (Zhang et al., 2018; Wang et al., 2016). Sections of testicular tissue (5 μm) were prepared and subjected to antigen retrieval and immunostaining as previously described (Wang et al., 2016). Briefly, sections were first blocked with normal goat serum in PBS, followed by incubation with primary Abs (Table S1; 1:100 in PBS-0.5% Triton X-100; Bioss Co. Ltd. Beijing, PR China) at 4^o^C overnight. After a brief wash, sections were incubated with an Alexa 546-labeled goat anti-rabbit secondary Ab (1:100 in PBS; Molecular Probes, Eugene, OR) at RT for 30 min and then counterstained with 4',6-diamidino-2-phenylindole (DAPI). The stained sections were examined using a Leica Laser Scanning Confocal Microscope (LEICA TCS SP5 II, Germany). Three animal samples from each of the control, NH_3_ and/or H_2_S treatment groups were analysed. The stained positive cells were count. A minimum of 1000 cells were counted for each sample of each experiment. Then the data were normalized to control.

**Statistical analysis**

Quantitative data were presented as the mean ± SEM. The data were statistically analysed by SPSS statistical software (IBM Co., NY). Statistical analysis of data was carried out by one-way analysis of variance (ANOVA), followed by LSD multiple comparison test. All treatment groups were compared with each other for every parameter. Differences were considered statistically significant if the p value was less than 0.05.
